# Observations on Scarlatina

**Published:** 1855-09

**Authors:** Henry Tweedy


### RECORD OF MEDICAL SCIENCE.

*Observations on Scarlatina.* By HENRY TWEEDY, M. D., &c.—There is no lack at present of able and useful contributions to our medical and surgical literature, but all must admit that it is of the first importance that practical men (many of whom have neither time nor taste for writing learned and lengthened essays) should be encouraged in every possible way to write as briefly and as plainly as they please upon points of practice not generally adopted, and the correctness of which has been proved in perhaps hundreds of unpublished cases.

The following observations upon scarlatina shall be confined to three points of practice:—

1ST. EARLY PURGATIVES.
2D. COLD DRINKS.
3D. THE APPLICATION OF NITRATE OF SILVER.

I have had extensive opportunity of witnessing and treating eruptive diseases when on different occasions they severally assumed the form of an epidemic in Dublin. Of scarlatina, which is justly considered the most formidable, I do in truth declare that, to the best of my belief, not a single case, for the last ten years, proved fatal of those which I had seen within twenty-four hours of the commencement of the attack, and which had not previously been put under treatment. Humanly speaking, the cause of such success has been that I have long held in deep abhorrence an *early* aperient in *any* form of eruptive disease. I state it advisedly as my opinion, and one not hastily formed, that a smart purgative draught is in itself sufficient to convert a case of mild scarlatina into a malignant, and, in all probability, a fatal one. To my mind it is not difficult to assign a reason as to how and why this should be so.

The primary stage of every disease, whether medical or surgical, is inflammatory, and this supposes the diminution—in some instances, the almost entire suspension—of all the secretions. The mucous membranes, which in health are always moist, become in the early stage of fever, and more especially the eruptive fevers, dry, rough, and parched. An irritating purgative which, in hundreds of cases, forms the groundwork of the treatment, increases considerably this state of things, and therefore should by all means be avoided; whereas medicines which tend to relieve the skin, subdue the fever, and restore the secretions, will, in almost every instance, cause the bowels to act freely and naturally.—Should such fail to be the case, a mild aperient, about the conclusion of the third day, will seldom fail in accomplishing all the indications required.

I consider this point one of great practical importance, involving the lives of many of our fellow-creatures. I have for years felt it my duty to warn parents, guardians, and proprietors of schools, &c., against the objectionable and most dangerous practice so often adopted, of giving aperient medicines during the prevalence of any epidemic. They have, to say the least of them, in numerous cases, done irreparable harm, lessening considerably, if not altogether destroying, all hopes of a good and complete recovery.

I cannot say that in one solitary case for the last ten years I ever ordered a particle of aperient medicine before the eruption was not only fully and fairly established, but further, beginning to decline; and I do positively assert that I never had cause to regret it. The bowels have, in many cases, been most satisfactorily relieved by medicines given with a totally different object, and in all the patients did well.

The medicine of all others on which I rely most, and the efficacy of which, in many diseases, is far greater than is generally known, is ipecacuanha. For a strong robust child of 10 or 12 years of age, I should order the following mixture :—

℞ Aq. distil. ℥vii.
Pulv. ipecac. gr. vi.
Syrupi croci ℥j. M.

One tablespoonful to be taken every second hour. The dose of the medicine should be increased or diminished in proportion to the age of the patient and the effect produced.

COLD DRINKS.

In connection with eruptive and inflammatory fevers generally, it may be well to mention that the objection entertained by many to cold water as a drink is, in my opinion, wholly untenable. I cannot possibly say in how many cases of scarlatina, measles, and other eruptive diseases, whether in adult or child, I have given the attendants full permission to give the patients cold water to any extent desired by them. Suffice it to say, that for at least ten years it has been, with perhaps one or two exceptions, my invariable rule. I am thankful to say, that not a solitary case has occurred which caused me to regret it. On the contrary, it has always proved not only a safe, but, in my opinion, the most useful, and certainly the most agreeable drink I could possibly employ.

While strongly advocating the propriety of giving patients as much cold water as they could possibly desire during the inflammatory stage of fever, I am not to be supposed to say that I should *order* every (or any) patient to drink quantities of this fluid against his will: quite the contrary. There are to be found some, especially in the upper ranks of life (I do not think I ever saw one in the lower), who, whether in sickness or in health, have an intolerable aversion to cold water as a drink. I have before my mind the names of some such, who have repeatedly acknowledged to me that never in their lives did they drink one glass of plain cold water at a time. With such I did not press the remedy, and each did well taking large quantities of warm drinks to assuage their intolerable thirst.

As there are physicians favorable to early purgatives, so there are those hostile to cold drinks in the first stage of eruptive fevers. My observation of the effects of both remedies in a large number of cases has led to the following conclusions :—

1st. A case may occur in which a purgative might be called for as the foundation of treatment in early fever, but I must ever maintain that such case is the exception, and not the rule.

2d. Cases may and do occur, in which early purgatives in fever do no apparent mischief; on the contrary, the patients progress satisfactorily.

3d. Constant observation forces upon me the conviction, that most serious consequences are produced by purgatives in the early stage of eruptive disease, eventuating in the most startling results, which, in my opinion, might have been avoided by other and milder treatment.

4th. The choice of warm whey, &c., or cold water, may safely be left with the patient. In nineteen cases out of twenty, the latter will be preferred. Gratifying the patient in the matter has never disappointed me.

### Nitrate of Silver.

The third consideration with reference to scarlatina, which I desire briefly to advert to, is the Application of Nitrate of Silver to the Fauces.

I believe no physician doubts the efficacy of this remedy, but certain I am that many practitioners might nearly, if not altogether, as well neglect this most useful and powerful agent, as use it as they do. There are many who never apply nitrate of silver to the throat in any other way than by means of a camel's hair pencil. Now, nothing could shake me in my conviction, that hundreds of cases of scarlatina have proved fatal from this cause alone. I have seen ulcerated sore throats, as severe as perhaps any which the records of medicine can afford, and which had brought the patients suffering from them to the very confines of the grave. Had I nothing better at hand than a camel's-hair pencil for applying a caustic solution, to a moral certainty they would all have terminated fatally.

The right mode, in my judgment, for employing this great (I might almost say only) remedy in this extreme case, in which life is all but extinct, is to get a long probe, round which should be rolled a piece of lint. This should be allowed to stand for about one minute in a twenty-grain solution of nitrate of silver. A spatula or spoon having been placed on the tongue to depress it, the probe should be passed low down into the throat, the interior of which should be cleverly rubbed all round. Those who have practised this in the way described have had their hearts gladdened by the wondrous change in a moment produced. A sponge attached to a rod of whalebone has been used and recommended by many. This, though infinitely preferable to a camel's-hair pencil, is open to objections :—1st. The same sponge should not be used (though I have seen it done) for more than one patient, and we often meet with two, three, or even more at the same time, and in the same house. 2d. There is a difficulty in having the sponge kept clean. 3d. The sponge is ten times more disagreeable to the patient; and lastly, it does not answer the purpose so well.

I know of no disease, the recoveries from which have astonished and rejoiced me so much as scarlatina. No case should be despaired of, or left without the most vigilant care, until life became wholly and unmistakably extinct. I feel perfectly satisfied that there are many practitioners who have reason (upon a review of past experience of this disease above all others) bitterly to regret the course they pursued —

Like croup, it runs through its stages quickly. To neglect proper treatment, and that at the right moment, or to employ injurious means, is an error never to be remedied.

Some time since I was attending a fine boy, aged seven years, in Mountjoy-square. He labored under malignant scarlatina. The case was one demanding the utmost care and attention, it being as unpromising—I might perhaps say as hopeless—a case as any I had ever seen. Having had occasion to pass the house at a very late hour of the night, I paid my little patient an unexpected visit. The person who opened the hall-door announced to me her belief that the child was dead. On entering the room I beheld the mournful sight of a fond father with what he considered to be his dead son lying in his lap. The scene was one not soon to be forgotten. On a very close examination, I could clearly perceive that the child was still alive, but no more. One drop of fluid he could not swallow, and his respiration was almost imperceptible. I instantly applied the nitrate of silver in the manner I have described; not, I must say, with much, (if any) expectation of success. The effect was literally miraculous. An immense quantity of mucus and lymph adhered to the lint; he immediately breathed freely, before I left the house drank cold water, and in the providence of GOD made a quick and complete recovery.

This is a case of peculiar interest, pointing out distinctly two important points—viz., 1st. The extreme value of nitrate of silver when properly applied. 2d. The physician's duty in never turning his back upon his patients, and saying no more can be done, so long as the smallest appearance of life remains.

A short time since I attended a family in North Richmond street, who have suffered severely from scarlatina. The mother took the disease from her children, four or five of whom were ill together. With the exception of one, the cases, though far from being mild, were not alarmingly severe. In the eldest, a handsome girl, about 11 years of age, the disease assumed a malignant type. On the night of the fourth or fifth day, I was sent for in great haste, and was informed by the messenger that my patient was dying. On entering the room I had good reason for fearing that what I had heard was but too true. Her face was absolutely purple; her respiration so painfully difficult that she required to be propped up with pillows in her bed; an intelligible word she could not utter, and swallowing was quite out of the question. I lost not a moment in applying the caustic solution, as in the last case mentioned, and with similar and equally happy results. Her recovery was everything that could be desired, and she is now, I am thankful to say, a healthy young lady, full of life and spirits.

I do not presume to say that the man who adopts the foregoing treatment must necessarily succeed in every or in any case; but this much I must say, that in my experience it has proved successful, and I confidently believe that none who adhere to it will have reason to regret it.

*Dublin Medical Press.*

